# Colorimetric Thermography by a Long‐Infrared Dual‐Band Metalens

**DOI:** 10.1002/advs.202408683

**Published:** 2024-11-19

**Authors:** Zhendong Luo, Peng Zhang, Huwang Hou, Yiming Li, Binzhao Li, Yanji Yi, Lianjie Xu, Ting Meng, Zihan Geng, Mu Ku Chen, Yang Zhao

**Affiliations:** ^1^ CAS Key Laboratory of Mechanical Behavior and Design of Materials Department of Precision Machinery and Instrumentation University of Science and Technology of China Hefei 230026 China; ^2^ Department of Electrical Engineering City University of Hong Kong Kowloon Hong Kong SAR 999077 China; ^3^ CAS Key Laboratory of Mechanical Behavior and Design of Materials Department of Modern Mechanics University of Science and Technology of China Hefei 230022 China; ^4^ Institute of Data and Information Tsinghua Shenzhen International Graduate School Tsinghua University Shenzhen Guangdong 518071 China; ^5^ State Key Laboratory of Terahertz and Millimeter Waves City University of Hong Kong Kowloon Hong Kong SAR 999077 China; ^6^ Key Laboratory of Precision Scientific Instrumentation of Anhui Higher Education Institutes University of Science and Technology of China Hefei 230022 China; ^7^ State Key Laboratory of Fire Science University of Science and Technology of China Hefei Anhui 230027 China

**Keywords:** colorimetric thermography, dual‐band metalens, emissivity, infrared detection

## Abstract

Infrared (IR) radiation thermography is extensively utilized in diverse fields due to its non‐contact capability. Nevertheless, its effectiveness is often compromised by the significant emissivity variations among different objects, limiting its application to specific setups or focused object types. Colorimetric thermography is introduced as an alternative emissivity‐independent method of radiation thermometry. This technique involves measuring radiance across two or more spectral bands and calculating the object's temperature based on the signal ratio, thereby mitigating emissivity effects under certain conditions. However, this method has the trade‐off of necessitating bulky optical systems, complex filter imaging configurations, and sensor structures. To meet the requirements of IR thermography for compact structure, lightweight design, and customizability, a dual‐band metalens is developed for the IR colorimetric thermography. The central wavelengths targeted are 9.5 and 12.5 µm. The dual‐band IR imaging by the fabricated dual‐band metalens is demonstrated, and the colorimetric thermography of low‐emissivity objects is performed without presetting emissivity values. This approach significantly eliminates measurement errors associated with emissivity by an average of 50.16% across a temperature range of 60–180 °C. This innovation paves the way for dynamic and multi‐target thermography using compact IR systems in complex environments.

## Graphical abstract

To meet the requirements of infrared (IR) thermography of compact components, a dual‐band metalens is developed for the IR colorimetric thermography. This approach significantly eliminates measurement errors associated with emissivity by an average of 50.16% across a temperature range of 600–180 °C, paving the way for dynamic and multi‐target thermography using compact IR systems in complex environments.

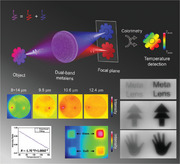

## 1 Introduction

Infrared (IR) thermography has the advantages of non‐contact and fast measurement and has become a widely used method in various fields, such as medical diagnosis,^[^ ^1^, ^2^, ^3^, ^4^ ^]^ industrial inspection,^[^ ^5^, ^6^ ^]^ and safety monitoring.^[^ ^7^ ^]^ Emissivity, *ε*, is a crucial parameter for accurate IR temperature measurement as it determines the object's radiation along with temperature. However, constrained by the substantial emissivity variety among different materials in the real world, conventional IR detection devices require the emissivity of the measured object to be provided in advance. Consequently, achieving accurate IR temperature measurement becomes challenging in cases where emissivity is unpredictable, especially in dynamic and multi‐target detection.

A viable solution to this issue is colorimetric thermography, a so‐called emissivity‐free radiation temperature measurement method.^[^ ^8^, ^9^, ^10^, ^11^ ^]^ The principle is to measure the radiance within two or more spectral bands and derive the object temperature from the signal ratio while eliminating the influence of the emissivity under certain conditions. There are many methods to realize IR spectrum detection, including the use of splitters and filters, multispectral sensors,^[^ ^12^, ^13^ ^]^ and micro‐optical resonators.^[^ ^14^ ^]^ Fabry–Perot structure composed of splitters and movable mirrors can be used to select wavelength, commonly known as the Fourier infrared spectrometer, and a rotating filter disc in front of the detector is also a solution. Furthermore, multilayer structures have been explored to achieve on‐chip multispectrum capabilities, and many researchers have done outstanding work in this area. Maier, Thomas et al. proposed an integrated solution for wavelength‐tunable absorption control in microbolometers using an absorbing metamaterial.^[^ ^12^ ^]^ Han et al. introduced a planar multimode antenna design capable of significantly enhancing the wavelength selectivity of a microbolometer.^[^ ^13^ ^]^ Wang et al. designed a fully tunable coupled absorption filter for thermal detectors using micro Fabry‐Perot resonators.^[^ ^14^ ^]^ However, all these approaches necessitate additional optical components or intricate sensor structures, resulting in a cumbersome system or expensive and challenging detector fabrication processes.

**Figure 1:**
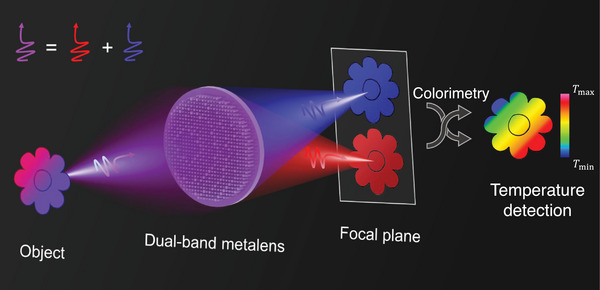
Schematic of dual‐band metalens imaging principle. After being imaged by designed metalens, two images of the object with different spectra will be formed in the focal plane. The temperature of the object can be obtained by colorimetry.

Recently, with the advantages of compact footprint, versatility, and custom design, metalens has been researched a bunch in many applications,^[^ ^15^, ^16^, ^17^, ^18^, ^19^, ^20^, ^21^, ^22^ ^]^ including polarization‐selective imaging in foggy conditions,^[^ ^23^ ^]^ ultrahigh numerical aperture and super‐resolution focusing,^[^ ^24^, ^25^, ^26^ ^]^ and dispersion control.^[^ ^27^, ^28^, ^29^, ^30^, ^31^ ^]^ Compared to traditional methods, metalenses offer a novel solution for achieving spectrum detection while ensuring a compact system and a simple fabrication process. In this work, we designed and fabricated a dual‐band wavelength‐selective metalens to realize IR colorimetric thermography with the central wavelengths of 9.5 and 12.5 µm, respectively. As shown in **Figure** **1**, the measured object is imaged through our dual‐band metalens, and two images with different spectra are formed on the focal plane. The temperature of the object can be obtained by colorimetry calculation. Dual‐band imaging with fabricated dual‐band metalens is demonstrated, and the colorimetric thermography of low‐emissivity objects is performed without presetting emissivity values. Our method eliminates the measurement errors caused by the emissivity of objects remarkably while keeping the optical system simple and compact.

## 2 Results

### 2.1 Principle of Colorimetric Thermography

According to Planck's law, the spectral exitance of electromagnetic radiation emitted by a black body can be written as

(1) $$M_{b}\;\;\;\left(\lambda ,T\right)=\frac{2hc^{2}}{\lambda^{5}\left(e^{hc/k\left(\lambda T\right)}-1\right)}$$

where *h* is the Planck constant, *c* is the speed of light, and *T* is the body temperature. When $hc/k{\left(\lambda T\right)}^{\prime \prime }1$, it can also be written as

(2) $$M_{b}\;\;\;\left(\lambda ,T\right)=\frac{2hc^{2}}{\lambda^{5}e^{hc/k\left(\lambda T\right)}}\;$$

After taking emissivity and system transmittance into account, the spectral excitance can be described as

(3) $$M\;\;\;\left(\lambda ,T\right)=\varepsilon \left(\lambda ,T\right)*M_{b}\left(\lambda ,T\right)*F\left(\lambda \right)$$

where *F*(λ) is the system transmittance (including the spectral response of infrared sensor, spectral transmittance of metalens, and atmospheric window). Thus, the radiation intensity received by sensors can be obtained after integration, which is

(4) $$M\;\left(T\right)=\int M\left(\lambda ,T\right)d\lambda =\int_{\lambda_{\mathrm{start}}}^{\lambda_{\mathrm{end}}}M_{b}\left(\lambda ,T\right)*\varepsilon \left(\lambda ,T\right)*\;F\left(\lambda \right)d\lambda$$

where λ_start_ ≈ λ_end_ is the operating bandwidth of the sensors (generally 8–14 µm for long‐wave infrared sensors).

**Figure 2:**
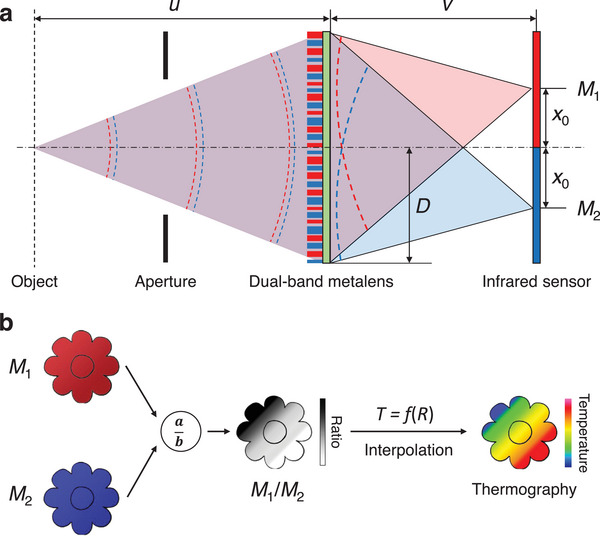
Operating principle of the colorimetric thermography. a) The dual‐band metalens encodes the phase profiles of two thin lenses in a single aperture. As wide‐band light passes through the metalens, it imposes distinct manipulations on the light components corresponding to different wavelengths. These two effective lenses maintain the same in‐focus distances but exhibit distinct off‐axis alignments (+ *x*_0_, −*x*_0_), creating two adjacent images on the separate parts of a single infrared sensor (red and blue). The intensities of these two images are recorded as *M*_1_ and *M*_2_. b) From a pair of input images, two straightforward calculations are performed to determine the temperature at each pixel, generating a final thermographic image. The process involves obtaining the intensity ratio of the corresponding pixels from the two input images, followed by deriving the temperature through interpolation.

As shown in **Figure** **2**a, when wide‐band light passes through the metalens, it imposes distinct manipulations on the light components corresponding to different wavelengths. With the imaging of the dual‐band metalens, two adjacent images are formed on separate parts of a single infrared sensor. The intensities of these two images are recorded as *M*_1_ and *M*_2_. For an ideal gray body, the emissivity of which is a function of temperature only, the intensity ratio of the two images after metalens imaging can be written as

(5) $$\begin{array}{ccc} R\;\;\;\left(T\right) & & =\frac{M_{1}\left(T\right)}{M_{2}\left(T\right)}=\frac{\varepsilon \left(T\right)*\int_{\lambda_{\mathrm{start}}}^{\lambda_{\mathrm{end}}}M_{b}\left(\lambda ,T\right)*F_{1}\left(\lambda \right)d\lambda }{\varepsilon \left(T\right)*\int_{\lambda_{\mathrm{start}}}^{\lambda_{\mathrm{end}}}M_{b}\left(\lambda ,T\right)*F_{2}\left(\lambda \right)d\lambda }\\ & & =\frac{\int_{\lambda_{\mathrm{start}}}^{\lambda_{\mathrm{end}}}M_{b}\left(\lambda ,T\right)*F_{1}\left(\lambda \right)d\lambda }{\int_{\lambda_{\mathrm{start}}}^{\lambda_{\mathrm{end}}}M_{b}\left(\lambda ,T\right)*F_{2}\left(\lambda \right)d\lambda }\; \end{array}$$

where *M*_1_(*T*) and *M*_2_(*T*) are the image intensity, *F*_1_(λ) and *F*_2_(λ) are the spectral transmittance of the two focal spots.

After extracted outside the integral, the emissivity can be eliminated by dividing the fraction above and below, as shown in Equation (5). As a result, the object's temperature can be detected directly by calculating the intensity ratio of its two images, and there is no interference from emissivity. It is evident that a mapping relationship exists between the ratio and temperature with certain *F*_1_(λ) and *F*_2_(λ), and this relationship curve *T* = *f*(*R*) can be derived experimentally. Therefore, with the relationship curve, a final thermographic image can be generated from a pair of input images through two direct calculations. The calculations involve obtaining the intensity ratio of the corresponding pixels from the two input images and deriving the temperature through interpolation, as depicted in Figure 2b. According to Gray Body Radiation Theory, most objects in the real world can be approximated as gray bodies. Therefore, this method is suitable for most real objects, with slight errors attributed to emissivity.

### 2.2 Design of Dual‐Band Metalens

Metalens, composed of subwavelength phase‐control nanostructures, can manipulate the wavefront of incident lights. With appropriate structure and parameter design, the phase controlling of every unit is independent, which is called locality.^[^ ^32^ ^]^ Spectral tailoring can be achieved in metalenses by spatial multiplexing.^[^ ^31^, ^33^ ^]^ Considering the atmospheric long‐wave infrared (LWIR) window of 8 ≈ 14 µm, the wavelength of *λ*_1_ = 9.5 µm and *λ*_2_ = 12.5 µm is set as the central wavelengths of the dual‐band metalens to balance the intensity of the two images. In order to function as a dual‐band imaging lens, the focal lengths of the two operating bands are 1 cm. The two foci are separated on the *x‐axis* by a distance of 1 cm. The metalens size is set to 2 cm in diameter. The phase modulation functions of the two single‐band metalenses can be written as:

(6) $$\Phi_{1}\;\;\;\left(x,y\right)=-2\pi n\left(\sqrt{{\left(x+x_{0}\right)}^{2}+y^{2}+f^{2}}-f\right)/\lambda_{1}$$

(7) $$\Phi_{2}\;\;\;\left(x,y\right)=-2\pi n\left(\sqrt{{\left(x-x_{0}\right)}^{2}+y^{2}+f^{2}}-f\right)/\lambda_{2}$$

where Φ_1_(*x*,*y*) and Φ_2_(*x*,*y*) are the phases at position (*x*, *y*), *x*_0_ = 0.5 cm is the deviation of focal spots from the *z*‐axis.

**Figure 3:**
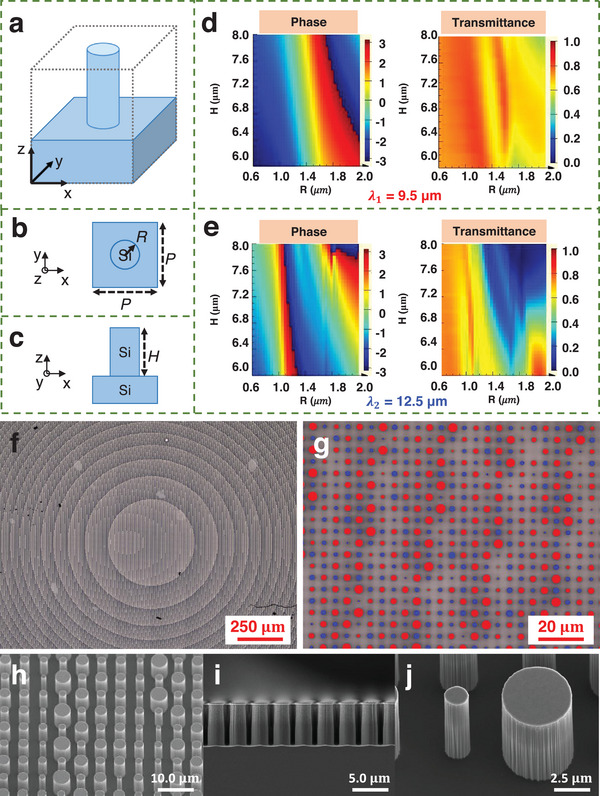
Design and characterization of dual‐band metalens. a) Silicon pillar on a square lattice silicon substrate. b) *x‐y* view and c) *x‐z* view of the unit cell. The lattice period of unit cells is *P*, while the radius and the height of pillars are *R* and *H*. d) The phase modulation map and the transmittance map of the unit cell at λ_1_ = 9.5 µm. e) The phase modulation map and the transmittance map of the unit cell at λ_2_ = 12.5 µm. f,g) Optical microscope image of the metalens at different magnifications(f: 10×;g: 100×). g) marks the pillar array corresponding to the two working wavelengths in red and blue colors. h, i, and j) SEM image of the metalens. (h: tilted view; i: cross‐section view; j: zoomed‐in tilted view.).

Silicon pillars on square lattice silicon substrate are selected as the unit cells here to achieve high transmittance and polarization independence of metalens.^[^ ^34^ ^]^ As shown in **Figure** **3**a–c, the lattice period of unit cells is *P*, while the radius and the height of pillars are *R* and *H*. According to Nyquist theorem, when designing a flat lens,^[^ ^15^ ^]^ the spatial discretization of the phase profile between adjacent unit cells satisfies the required sampling criterion

(8) $$P\leq \;\frac{\lambda }{2NA}=\frac{\lambda }{2nsin\theta }\;$$

where *n* (= 1) is the refractive index of air and θ is the deflecting angle of the transmitted light. Considering the metalens design parameters (diameter *D* = 2 cm and focal length *f *= 1 cm, *NA *= 0.71) and the shorter design central wavelength 9.5 µm, it can be calculated that *P* ≤ 6.36 µm. The smaller the *P*, the higher the sampling frequency. However, when *P* is too small, the radius can only be adjusted within a narrow range due to the line width limitations of the machining process. Achieving adequate phase coverage under this condition necessitates taller structures with a larger depth‐to‐width ratio. Considering these factors, a unit cell period *P* of 5 µm was selected to meet the sampling requirements while avoiding a large depth‐to‐width ratio. The property maps of unit cells with varying *R* and *H*, as shown in Figure 3d,e, are obtained numerically by the 3D finite‐difference time‐domain algorithm with the commercial software package FDTD solutions provided by *Lumerical Solutions, Inc*. To meet the minimum line width of 1 µm in processing and ensure the precision of the nanopillars, the variation range of *R* is selected to be 0.6 ≈ 2 µm (diameter *D* 1.2 ≈ 4 µm). It is important to note that the selection of *H* and *R* must ensure phase coverage exceeding 2π and pursue a high average transmittance to meet the functional requirements of an imaging lens. According to Figure 3d,e, when *H* is 7 µm, the nanopillars provide phase coverage exceeding 2π at both 9.5 and 12.5 µm incidence. However, when the height exceeds 7 µm, the transmittance decreases significantly at 12.5 µm incidence. Hence, the height *H* of 7 µm is selected. The phase control at different locations can be calculated based on Equations (6) and (7). Subsequently, the radius distribution of the nanopillars in the metalens can be determined by referencing the property map in Figure 3d,e. Here, we get two sets of nanopillar distributions at 9.5 and 12.5 µm, respectively. By spatially combining these distributions, the final layout of the dual‐band metalens is achieved (see Appendix, Section S1, Supporting Information). In light field simulation, the incident plane light of 8 ≈ 14 µm is focused into two spots on the focal plane, each with a full width at half maximum (FWMH) of 12 µm (see Appendix, Sections S2 and S3, Supporting Information).

Figure 3f,g are the optical images of the fabricated metalens. The results show that most of the pillars meet expectations and are comparable to the design dimensions. The scanning electron microscopy (SEM, Hitachi, SU8220) images of the fabricated metalens are shown in Figure 3h,g. The pillars with various sizes are well‐defined, displaying precise fabrication techniques with the hard mask pattern transfer and etching processes.

### 2.3 Imaging and Colorimetric Thermography

**Figure 4:**
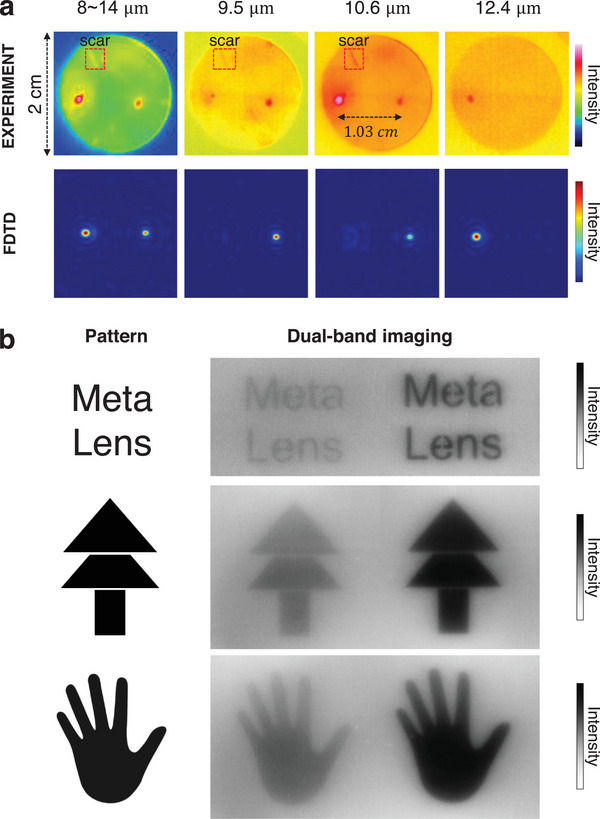
Bi‐focusing and dual‐band imaging. a) Experimental (top row) and numerical (bottom row) intensity profiles in focal planes at various incident central wavelengths (CW). Because of the different bandwidths of the filters in the experiment (CW: 9.5 µm, bandwidth: 0.5 µm; CW: 10.6 µm: bandwidth:1.5 µm; CW: 12.4 µm, bandwidth:0.25 µm), the color bar of the intensity is not uniform here. The scar was caused by an accidental scratch during the machining process, which is less visible in the fourth image due to the reduced peak difference of the color bar. b) Images of different patterns. Due to the spectral non‐uniformity of blackbody radiation and variations in system transmittance, some differences arise in the intensity of the two images.

Bi‐focusing and dual‐band imaging are demonstrated in the experiment, as shown in **Figure** **4**. Detailed experimental setups are provided in the Sections S4, S5 (Supporting Information). Filters of different bands are alternately inserted between the plane light and metalens to measure the distribution of focus only under various wavelengths. These filters are then removed in later experiments. Figure 4a shows that the measured and simulated light intensity profiles on the focal plane are in good agreement with each other. There is some deviation between the experimental and FDTD results in the third column (10.6 µm). These discrepancies are attributed to differences in the setup. The simulation assumes an ideal narrow‐band 10.6 µm incidence, whereas the filter used in the experiment has a relatively broad bandwidth, 1.5 µm. The measured distance between the two focal spots is 1.03 cm, which is close to the designed 1 cm. The light intensity shifts from the right focal spot to the left focal spot while the incident wavelength increases, indicating the realization of a wavelength‐selecting and focusing property in the long‐IR region. Dual‐band imaging of various patterns is depicted in Figure 4b. Two distinct images are captured on the infrared sensor, exhibiting some intensity differences from the theoretical result. This is due to other components of the system, besides the dual‐band lens, may contribute to some degree of wavelength filtering. But once the dual‐band metalens being inserted into a certain system, the overall system transmittance will remain stable.

**Figure 5:**
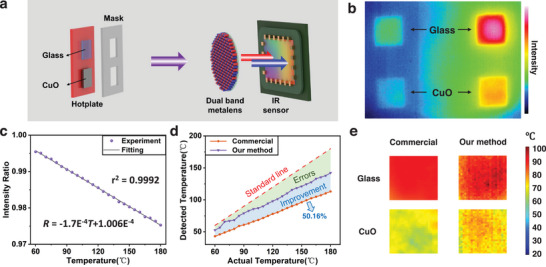
Experimental setup and results of colorimetric thermography. a) Experimental setup. b) Dual‐band imaging of glass and CuO plate. c) Experimentally measured intensity ratios and temperatures, using the glass plate as a blackbody due to its high and stable emissivity of 0.9. The fitting curve exhibits a strong linear relationship, evidenced by a high coefficient of determination, R^2 ^= 0.9992. d) Temperature detection of CuO plate without predicated emissivity by a commercial infrared camera (FLUKE Tix660) and our method. With our method, the detected temperature line is much closer to the standard line, and the error is remarkably reduced (purple area) by an average of 50.16% in the temperature range of 60 to 180 °C (indicated by the purple region). e) Temperature map captured by a commercial infrared camera (left) and derived from our method (right).

The relationship curve between temperature and intensity ratio is measured, and colorimetric thermography is performed on objects with low emissivity. The experimental setup, depicted in **Figure** **5**a, involves two sheets of copper oxide (CuO) and glass mounted on a hotplate, which serves as the infrared radiation source. The interface between the sheets and the hotplate is filled with thermal grease to ensure optimal thermal conduction. The temperature of the sheets can be regulated by adjusting the hotplate's temperature. A mask with two rectangular openings is positioned directly behind the radiation source, effectively shielding radiation emanating from the hotplate itself. Due to differing emissivities, 0.9 for glass and 0.51 for CuO, the two sheets, although at the same temperature, produce distinctly different thermal images in brightness, as shown in Figure 5b.

Here, we take the glass as a blackbody for its high and stable emissivity of 0.9, and the fitting curve is obtained by measuring its intensity ratio at various temperatures, as shown in Figure 5c. The fitting curve, *R* = −1.7E^−4^ *T*+1.006E^−4^, exhibits a strong linear relationship, evidenced by a high coefficient of determination, r^2^ = 0.9992. Due to the low emissivity, the temperature of CuO detected by the commercial infrared camera without predicated emissivity substantially deviates from the actual temperature, as indicated by the orange line in Figure 5d. With a fitting curve as a reference, the temperature of CuO can be calculated by interpolating its measured intensity ratio, as shown by the purple line in Figure 5d. It can be seen that, with our colorimetry method, the detected temperature line is much closer to the standard line. By calculating the area between the measured line and the standard line, which represents the degree of error, it can be seen that the error is remarkably reduced by an average of 50.16% (purple area in Figure 5d) within the temperature range of 60 to 180 °C. Using point‐to‐point colorimetry, the thermography of the two sheets is obtained, as depicted in Figure 5e, with the actual temperature recorded at 100 °C. Compared to commercial thermography, the temperatures of both the glass and CuO sheets measured with our method match their actual temperatures better (see detailed 3D temperature bar graph in the Section S6, Supporting Information).

## 3 Discussion

The response of traditional IR detectors can be affected not only by temperature but also by objects' emissivity and the systems' optical transmittance. In the real world, the emissivity of different objects varies greatly, posing challenges for dynamic detection or multi‐target detection in complex backgrounds. Colorimetric thermography can substantially reduce the errors caused by the emissivity of objects but simultaneously results in bulky optical systems or intricate sensor structures. In this work, a dual‐band metalens has been devised to achieve infrared colorimetric thermography while keeping the optical system simple and compact. IR imaging with the dual‐band metalens is demonstrated, and the colorimetric thermography of low‐emissivity objects without presetting emissivity is realized. By this colorimetric temperature measurement method achieved with fabricated dual‐band metalens, the measurement errors caused by the emissivity of objects are eliminated remarkably by an average of 50.16% in the temperature range of 60 to 180 °C.

We notice that there are still discrepancies compared to the actual temperature. One of the reasons is that glass used as a blackbody in the experiment is not an ideal blackbody. Consequently, the curve representing the relationship between temperature and intensity ratio measured deviates from the ideal curve. Another reason is that it is inevitably affected by the radiation reflected from the ambient due to its low emissivity. For large *ε* values, the reflection contributions of opaque objects are minimal. However, objects such as metals, which have very low emissivities, present challenges because the emitted radiance is low while the reflected radiance is high. At the same time, the edges of the sheet appear insufficiently sharp due to the limited alignment accuracy of the left and right images in our calculation. The detection accuracy is expected to be further enhanced with better blackbody calibration and corresponding image recognition algorithm, presenting a new opportunity for dynamic detection and multi‐target detection of compact IR detectors in complex environments.

## 4 Experimental Section

### Metalens Fabrication

A single crystal silicon substrate was cleaned and prepared for metalens fabrication. A 200 nm‐thick aluminum film was deposited as a hard mask layer by the electron beam evaporation (K.J. Lesker LAB 18). Considering the large aperture of the metalens, the maskless lithography (ATD 1500) was adopted here instead of the costly and time‐consuming electron beam lithography, which was used extensively in the fabrication of visible metalens. The minimum linewidth requirement of maskless lithography had already been taken into account when selecting the size of Metalens' unit cells. Maskless lithography for 30 min using a 4 mm writer head, followed by 1 min and 15 s of Inductively Coupled Plasma (ICP) etching (Oxford, Plasma System100 ICP180), transfered the designed layout and formed the hard mask on the aluminum film. The 7 µm‐thick silicon structural layer was etched for 5 min using a deep silicon etching system (Oxford, Estrelas 100) to ensure vertically etched sidewalls. Finally, the hard mask was removed by using the aluminum etchant, leaving the array of silicon unit cells with various radii. A detailed illustration of the fabrication process is provided in the Section S7 (Supporting Information).

## Conflict of Interest

The authors declare no conflict of interest.

## Supporting information



Supporting Information

## Data Availability

The data that support the findings of this study are available from the corresponding author upon reasonable request.
