# Supplementary material for: Colorimetric Thermography by a Long‐Infrared Dual‐Band Metalens
Source: Adv Sci (Weinh). 2024 Nov 19;12(2):2408683. doi: 10.1002/advs.202408683 (PMC11727133; doi:10.1002/advs.202408683)
Supplement: Supplementary file 1 — Supporting Information [file ADVS-12-2408683-s001.docx]

**Supplementary Information**

**Colorimetric** **thermography by an infrared dual-band metalens**

**Zhendong Luo^a^****^,d,#^, Peng Zhang^a,#^, Huwang Hou^b^, Yiming Li^a^, Binzhao Li^b^, Yanji Yi^a^, Lianjie Xu^a^,** **Ting Meng^a^, Zihan Geng^c^, Mu Ku Chen^d,e,*^, Yang Zhao^a,f,g,*^**

**^a^** **CAS Key Laboratory of Mechanical Behavior and Design of Materials, Department of Precision Machinery and Instrumentation, University of Science and Technology of China, Hefei 230026, China**

**^b^ CAS Key Laboratory of Mechanical Behavior and Design of Materials, Department of Modern Mechanics, University of Science and Technology of China, Hefei 230022, China**

**^c^ Institute of Data and Information, Tsinghua Shenzhen International Graduate School, Tsinghua University, Shenzhen, Guangdong 518071, China**

**^d^ Department of Electrical Engineering, City University of Hong Kong, Kowloon, Hong Kong SAR, China**

**^e^ State Key Laboratory of Terahertz and Millimeter Waves, City University of Hong Kong, Kowloon, Hong Kong SAR, China**

**^f^ Key Laboratory of Precision Scientific Instrumentation of Anhui Higher Education Institutes, University of Science and Technology of China, Hefei 230022, China**

**^g^ State Key Laboratory of Fire Science, University of Science and Technology of China, Hefei, Anhui 230027, P.R. China**

**This file includes:**

Section S1. Spatial multiplexing dual-band metalens

Section S2. Simulations on narrow-band IR light field propagation.

Section S3. Simulations on wide-band IR light field propagation.

Section S4. Experiment setup for bi-focusing

Section S5. Experiment setup for dual-band imaging

Section S6. Comparison of temperature detection between commercial and our method

Section S7. Metalens fabrication

**Section S1. Spatial multiplexing dual-band metalens**

With appropriate structure and parameter design, the phase controlling of every unit is independent. Thus, spectral tailoring can be achieved in metalenses by spatial multiplexing. As shown in Fig. S1, the studied dual-band metalens is constructed by interlacing unit cells separated from two single-band metalenses. Under the assumption of locality, the coupling between nanopillars can be neglected, and the incident light can be converged to respective focal spots at each design wavelength. The choice of central wavelengths has a decisive influence on the performance of temperature measurement and imaging. When the two operating wavelengths are too close to each other, the image intensity ratio resulting from temperature variations decreases, thereby reducing the sensitivity of the measurement. When the central wavelength is too close to the boundary of the atmospheric window, a significant portion of the light is blocked, reducing the signal received by the detector and increasing its susceptibility to noise interference. Therefore, considering the atmospheric long-wave infrared (LWIR) window of 8~14 μm, the wavelength of λ_1_ = 9.5 μm and λ_2_ = 12.5 μm are set as the central wavelengths of the dual-band metalens to balance the intensity of the two images.


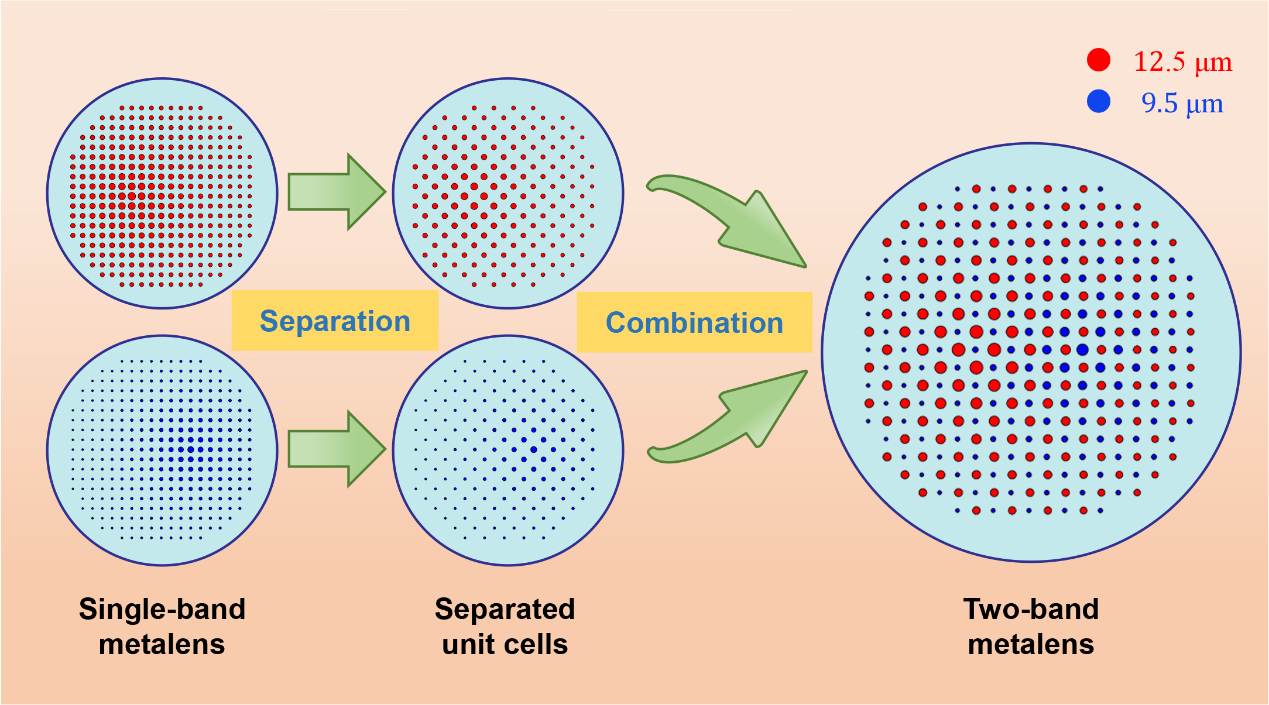


**Fig. S1.** Schematic of a dual-band metalens constructing process. By separating unit cells from two single-band metalenses and interlacing them together, a dual-band metalens can be obtained.

**Section S2. Simulations on narrow-band IR light field propagation.**

Narrow-band IR light field propagation is simulated for the designed metalens, and the results are shown in Fig. S2. For computational efficiency, a metalens model with a focal length and diameter 100 times smaller than the designed imaging metalens was used as an alternative in the simulations. Fig. S2(a) shows the light intensity distributions of the x-y plane at the focal plane (upper row) and the light field distributions of the x-z plane (lower row). With the variation of incident wavelengths, light intensity shifts from one focal spot to another. It can be seen that the light within a certain band near the designed central wavelengths will contribute to the intensity at the focal spots. The spectral transmittance of left and right focal spots and intensity distribution along the *x*-axis at different wavelengths were analyzed, as shown in Fig. S2(b,c). Given that the dual-band metalens comprises only 50% of the nanopillars from each single-band metalens, a 50% reduction in theoretical efficiency is inevitable. As shown in Fig. S2(b), the spectral transmittances of left and right focal spots are lower than 0.5 at their respective central wavelength.


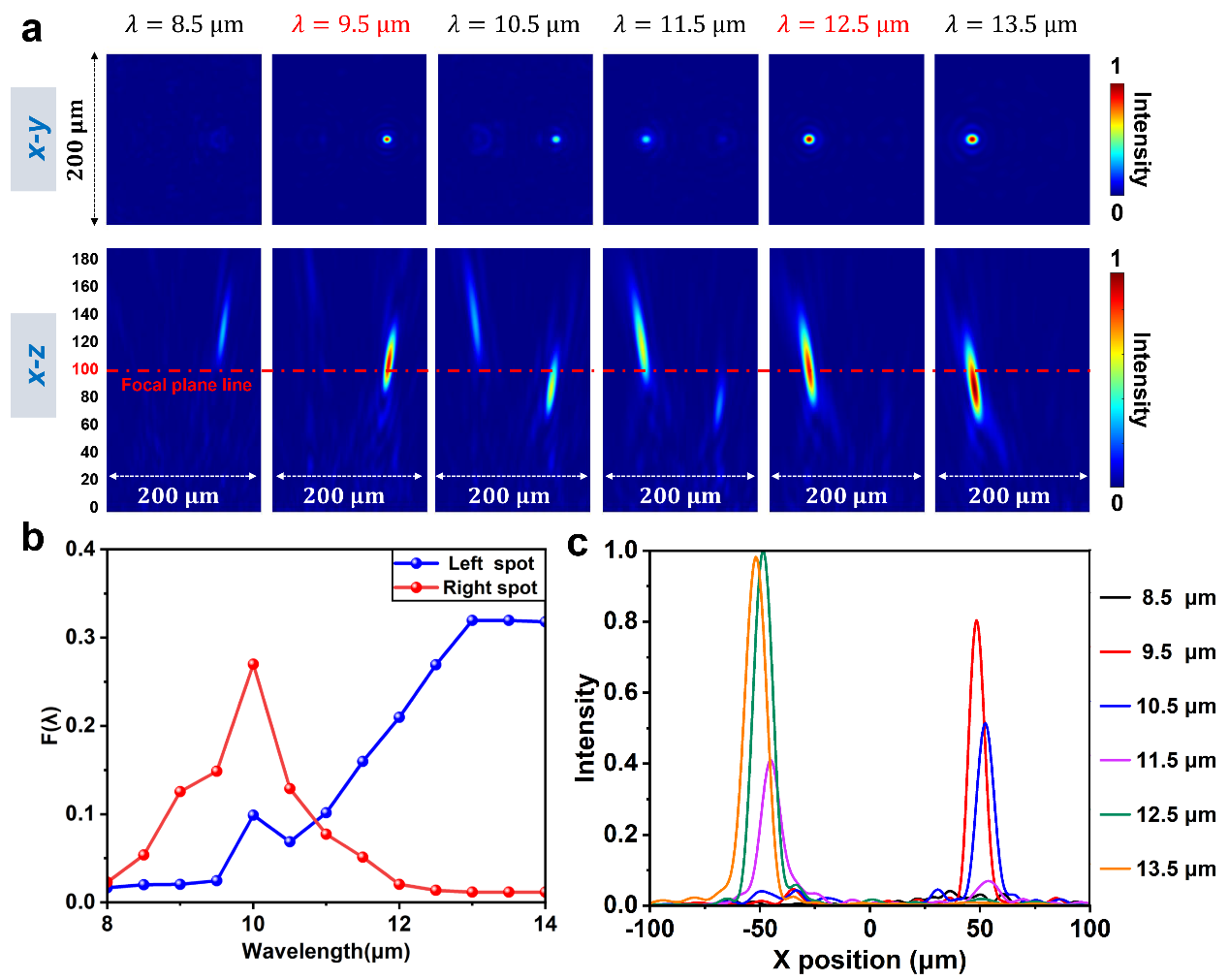


**Fig. S2.** Simulations on narrow-band IR light field propagation. (a) Intensity profiles of metalens in *x-y* planes (focal plane) and *x-z* planes at various incident wavelengths. The red line indicates the location of the design focal plane. (b) Spectral transmittance/efficiency of left and right focal spots. (c) Normalized intensity distribution along the *x*-axis under different incident wavelengths.

Fig. S3 presents the spectral response of the single-band metalenses. It can be observed that the spectral response of the dual-band metalens effectively combines the characteristics of the two single-band metalenses.


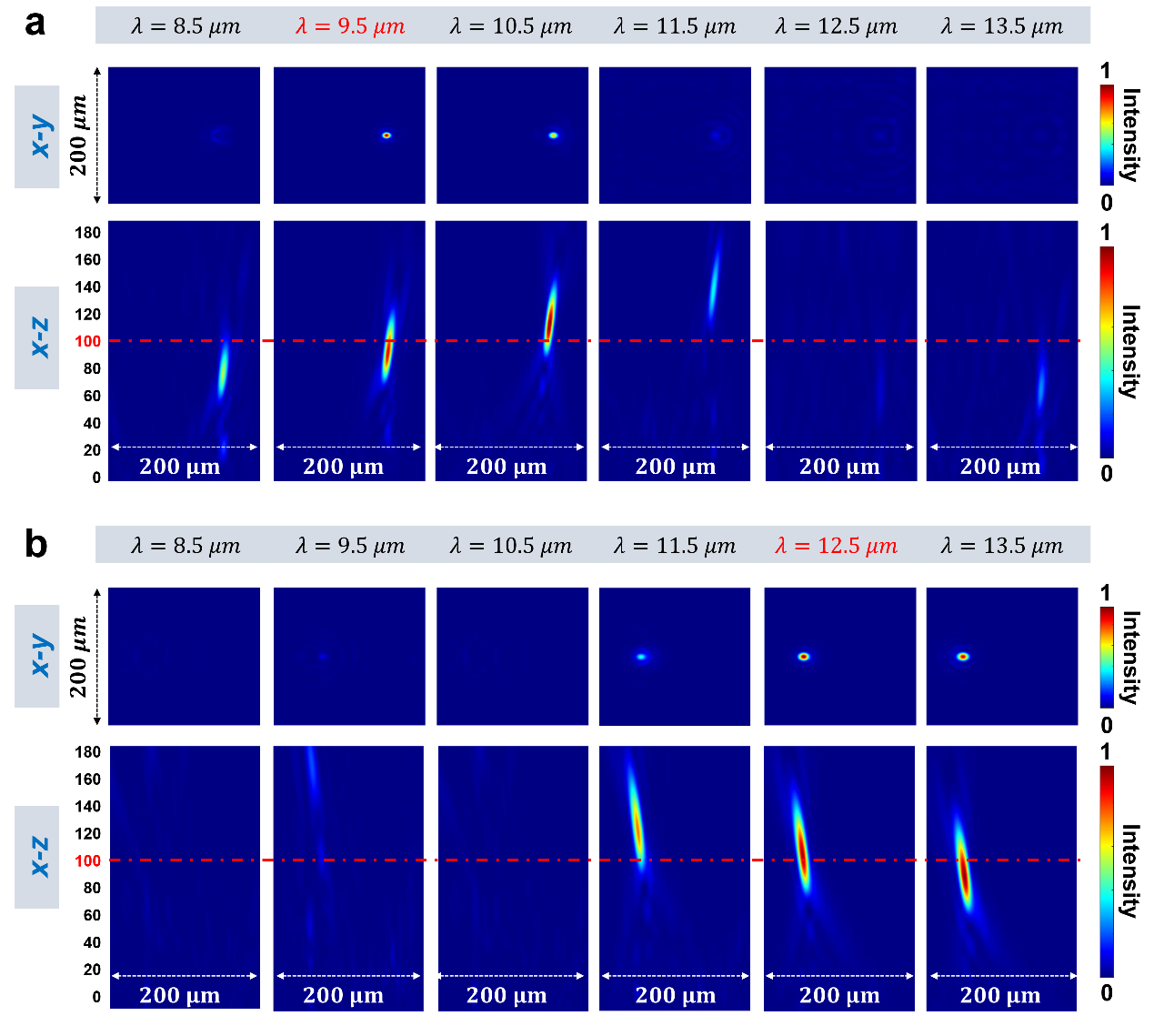


**Fig. S3.** Simulations on narrow-band IR light field propagation of single-band metalenses. Intensity profiles of metalens in *x-y* planes (focal plane) and *x-z* planes at various incident wavelengths. The red line indicates the location of the design focal plane. The central wavelengths of single-band metalenses are (a) 9.5 μm and (b) 12.5 μm, respectively.

**Section S3. Simulations on wide-band IR light field propagation.**

As shown in Fig. S4, the incident plane light of 8~14 μm is focused into two spots on the focal plane, each with a full width at half maximum (FWMH) of 12 μm and spaced 100 μm apart. Due to the achromatic, the diameter of the spot appears slightly larger than that observed in the narrow band. Although the efficiency reduction, the dual-band metalens effectively combines the spectral response characteristics of the two single-band metalenses and maintains a similar FWHM.


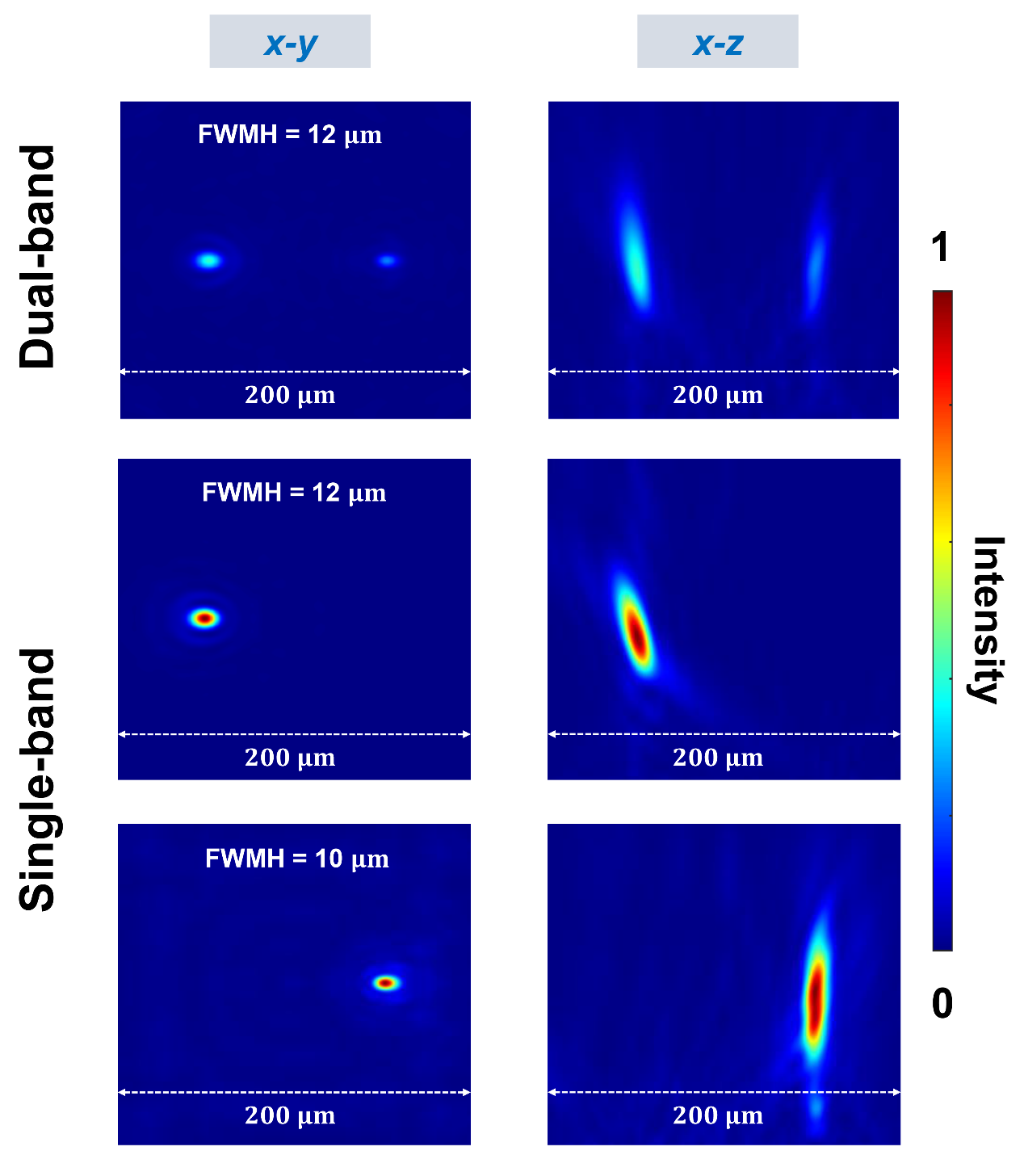


**Fig. S4.** Simulations on wide-band IR light field propagation. Intensity profiles of metalens in the *x-y* plane (focal plane) and *x-z* plane at a wide incident light of 8~14 μm.

**Section S4. Experiment setup for bi-focusing**

As shown in Fig. S5, the infrared light source comprises a hotplate, an aperture, and a long tube. The aperture serves to block excess light radiating from the hot plate, while the long tube ensures the parallelism of the emitted infrared light. Filters were alternately inserted between the plane light and metalens to measure the distribution of focus under various wavelengths, and was then removed in later experiments.


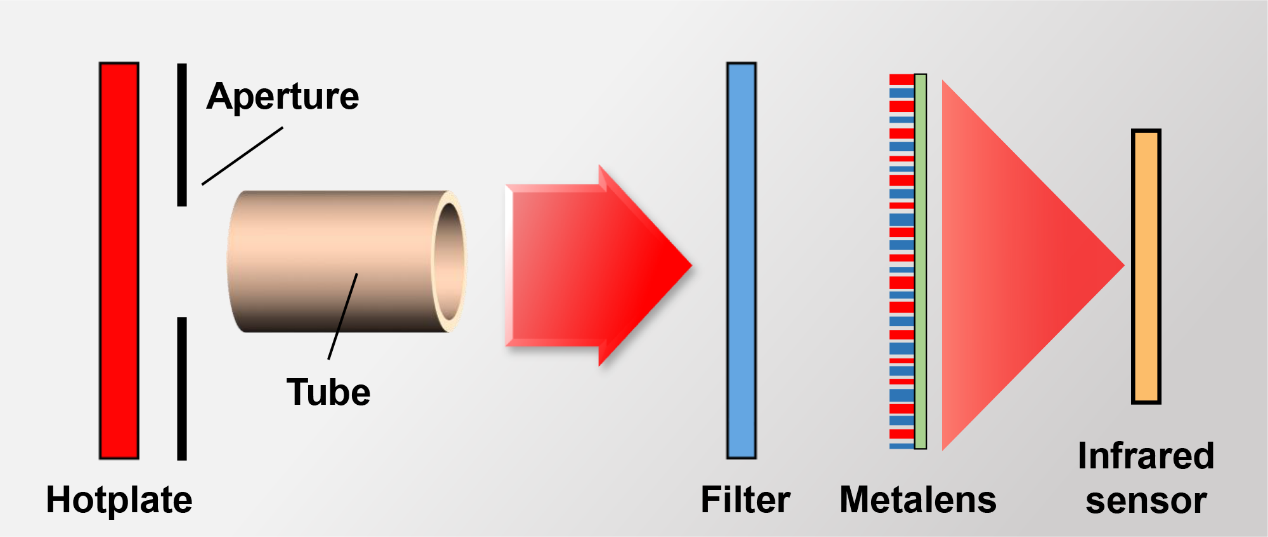


**Fig. S5.** Experiment setup for bi-focusing. Different filters are alternately inserted to control the wavelength of incident light (CW: 9.5 μm, Width: 0.5 μm; CW: 10.6 μm: Width:1.5 μm; CW: 12.4 μm, Width:0.25 μm).

**Section S5. Experiment setup for dual-band imaging**

Masks with patterns are alternately positioned directly behind the hotplate, as depicted in Fig. S6. Part of the pattern allows the transmission of infrared light, while the remainder blocks the radiation.


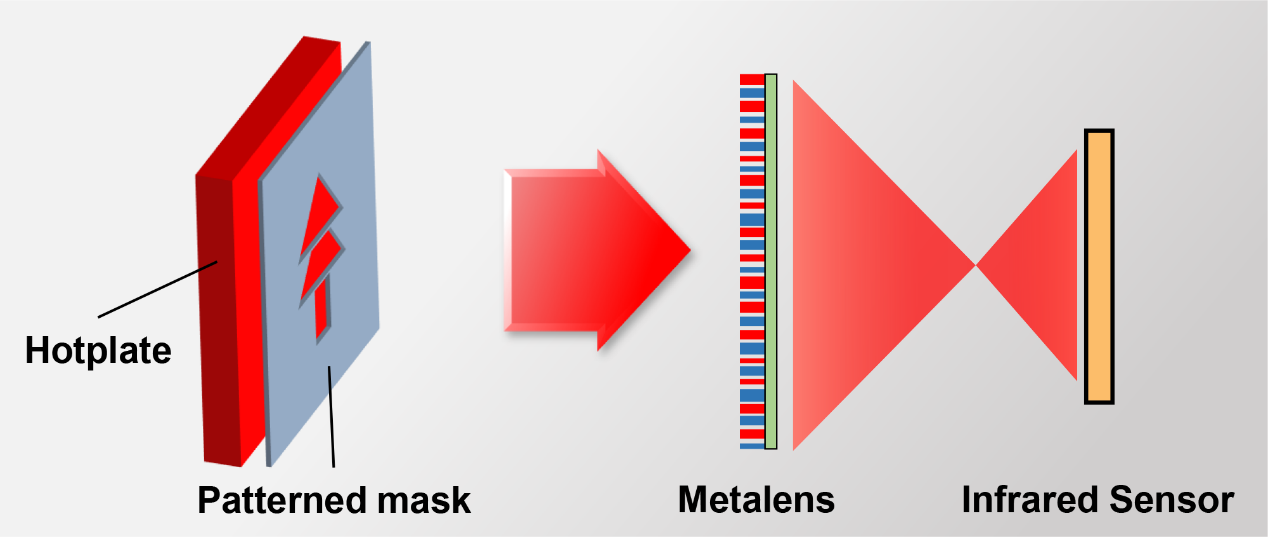


**Fig. S6.** Experiment setup for dual-band imaging. Patterned masks are employed to selectively filter infrared radiation.

**Section S6. Comparison of temperature detection between commercial and our method**

Fig. S7 presents the 3D temperature bar graph of glass and CuO from different views. Compared to commercial thermography, the temperatures of both the glass and CuO sheets measured with our method closely match their actual temperatures of 100 Celsius. However, due to the use of preliminary image alignment techniques, the results measured with our method exhibit more noise. This noise is expected to be mitigated by employing more accurate image alignment algorithms.


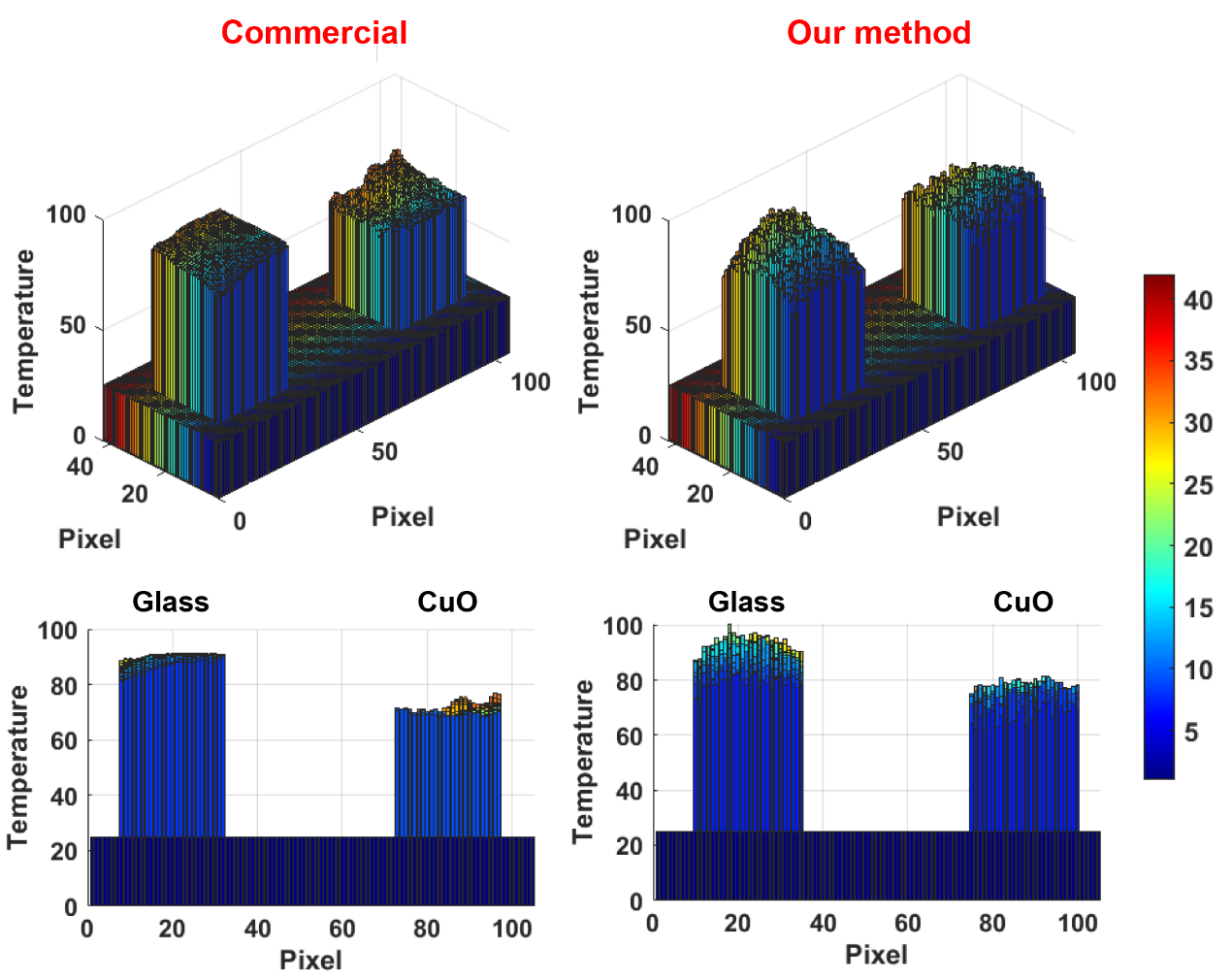


**Fig. S7.** 3D bar of the detected objects’ temperature. Left: commercial method results; right: our method results. Top row: slanted view; bottom row: flat view.

**Section S7. Metalens fabrication**

As depicted in Fig. S8, there are six steps in the metalens fabrication process: ⅰ). Deposition of the aluminum film by Electron Beam Evaporation. ⅱ). Photoresist coating. ⅲ). Patterning of photoresist using maskless lithography. ⅳ). The aluminum hard mask is etched by the inductively coupled plasma etching. ⅴ). Etching silicon to form the pillar array. ⅵ). Removal of the hard mask.


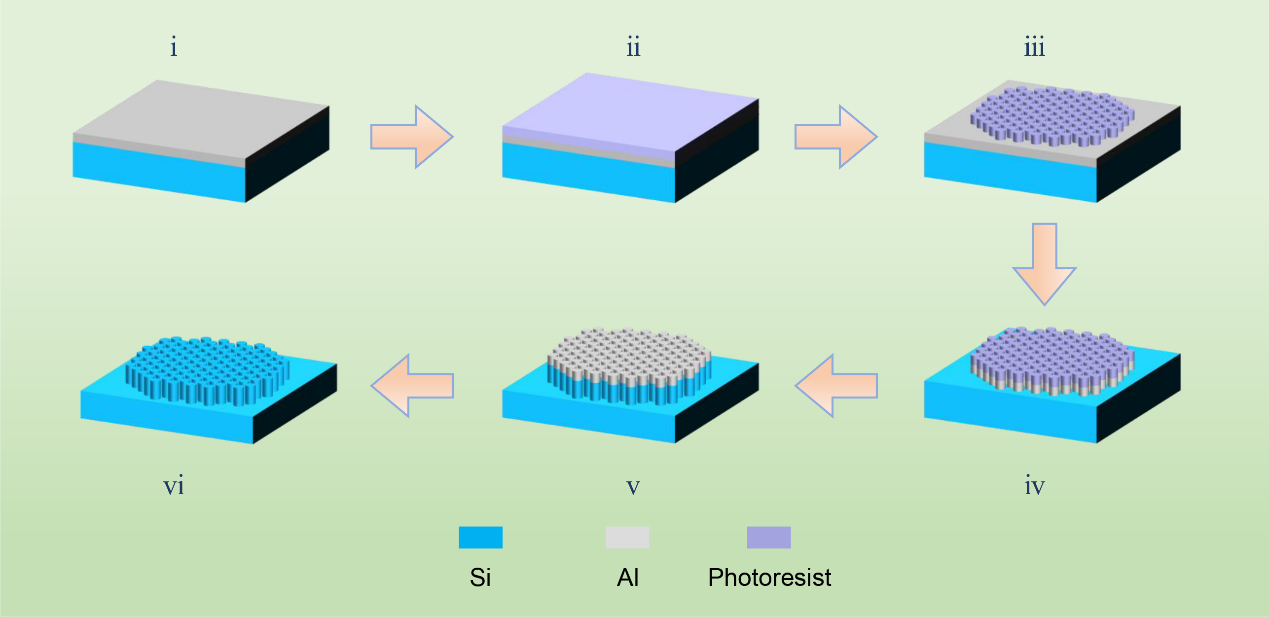


**Fig. S8.** Schematic showing the major steps of the fabrication process.
